# Reduction of Leaching Impacts by Applying Biomass Bottom Ash and Recycled Mixed Aggregates in Structural Layers of Roads

**DOI:** 10.3390/ma9040228

**Published:** 2016-03-24

**Authors:** Manuel Cabrera, Adela P. Galvin, Francisco Agrela, Manuel G. Beltran, Jesus Ayuso

**Affiliations:** Area of Construction Engineering, University of Cordoba, Cordoba 14071, Spain; manuel.cabrera@uco.es (M.C.); apgalvin@uco.es (A.P.G.); manugabel@gmail.com (M.G.B.); ir1ayuje@uco.es (J.A.)

**Keywords:** heavy metals, stabilization/solidification, leaching, bottom ash

## Abstract

This research is focused on analyzing the environmental pollution potential of biomass bottom ashes as individual materials, as mixtures manufactured with biomass bottom ashes and granular construction aggregates, and these mixtures treated with cement. For the environmental assessment of all of the samples and materials mentioned, the following leaching procedures have been performed: the compliance batch test of UNE-EN 12457-3:2003 for aggregates and bottom ashes; the column test according to NEN 7343:1994 for the mixtures prepared in the laboratory; and the tank test by EA NEN 7375:2004 for analyzing the behavior of mixtures after their solidification/stabilization with 5% cement. After the discussion of the data, the reduction of the pollution load of the most hazardous biomass bottom ashes after their combination with different aggregates can be confirmed, which implies their possible application in civil infrastructures, such as filler embankments and road construction layers, without negatively impacting the environment. In addition, the positive effect of the stabilization/solidification of the cement-treated mixtures with a reduction of the heavy metals that were released at the highest levels, namely As, Hg Cr, Ni, Cu, Se and Mo, was proven.

## 1. Introduction

In industrialized countries, it is expected that the future generation of electricity will be from the direct combustion of residues and wastes obtained from biomass [1]. Biomass boilers are a medium for efficiently combusting biomass. Boiler combustion processes are known to produce large amounts of bottom ash. For that reason, the primary concerns are ash storage, disposal and usage. The major inherent biomass bottom ash (BBA)-forming elements in biomass include Ca, Si, Al, Ti, Fe, Mg, Na, K, S and P [2,3]. Bottom ashes are composed of minerals that were either absorbed by the biomass or incorporated into the biomass during harvesting, and unburned organic matter. Although previous studies have demonstrated the mechanical aptitude of bottom ashes for minor constructive uses, their potential reuse in civil engineering works is determined by their chemical and physical properties [4].

Various researchers have investigated different processing methods to improve the mechanical properties of BBA for use in engineering applications. Due to the huge variety of biomass fuel sources with differing ash properties, the identification of the physical, chemical and environmental characteristics of ashes will provide valuable information as to the likely methods for optimizing their processing and reuse as secondary materials [5].

Currently, the construction industry uses fly ashes in different products, such as a cement replacement in concrete [6], for soil stabilization, as road base [7,8] and in cement production [9]. Additionally, bottom bed ashes from the combustion of forest biomass residues are used as substitutes for cement and natural aggregates in concretes without compromising their mechanical, chemical, and ecotoxic properties [10], as aggregates in rendering mortar formulations [11,12] and for civil infrastructures, proving their technical feasibility [13]. While much research has been conducted on bottom ash utilization, much remains to be discovered regarding the effective management of this material in order to lead to an environmentally friendly product that does not cause negative impacts on urban and rural surroundings.

Thus, the policy of increasing the use of renewable energy, driven by the Spanish government (Plan de Acción Nacional de Energías Renovables (PANER) 2011–2020), has caused a remarkable increase not only in the kilowatts (kW) produced by biomass combustion-based power plants, but also the tonnage of ashes produced annually [14]. Calculations put the potential biomass resources in Spain at 19,000 ktep (13,000 ktep corresponds to residual biomass, and almost 6000 ktep corresponds to energy crops), and this resource is considered a fundamental pillar for meeting the objectives pursued. This has caused the plant management, pollutant potential and disposal of the resulting ashes (fly and bottom ash) to become an important environmental and economic issue for the state government. Thus, in Spain, despite scientific advances, many tons of BBA are not revalued and deposited in landfill. Given its high availability, it is interesting to consider a feasibility study for the recovery of such waste in construction or civil engineering, and this is the main motivation for the present research. The potential utilization of BBA is influenced not only by its technical feasibility, but also by the contaminant content, such as heavy metals, and the possibility of their incorporation into the surrounding environment. Effective environmental monitoring, environmental assessment by means of leaching tests and protective actions are necessary to ensure that bottom ash (BA) disposal does not become an environmental hazard.

In that sense, previous researchers have demonstrated the reduction of the pollution load of different wastes by solidification/stabilization (S/S) as an effective environmental protective action that prevents and minimizes the release of the contaminant into the environment. Through the conversion of heavy metals into a less mobile form, Maschio *et al.* [15] replaced commercial cement with different percentages of biomass bottom ash, Barbosa *et al.* [16] demonstrated that the S/S materials have compressive strengths between 25 and 40 MPa and low emission levels of metals through leaching, and they were thus classified as non-hazardous materials. The most widely used S/S systems are cement-based materials due to their viability for forming durable monolithic materials that will not easily leach hazardous components under disposal conditions [17].

The present research study focuses on the BBA generated by three biomass combustion power plants and investigates the environmental challenges that increased bottom ash generation may cause as a consequence of the current state regulations. The presence of heavy metals in the leachates of tested BBA was evaluated by different leaching procedures to prevent significant impacts.

The contaminant load of heavy metals on the leachates of 30 BBA samples were characterized through a normalized compliance test. After detecting the most contaminated BBA, they were selected for the preparation in the laboratory of two types of mixtures of bottom ashes and aggregates: type 1 with natural aggregate (NA), and type 2 with recycled mixed aggregates (RMA). By performing a percolation test, the reduction of heavy material release was observed. Finally, the monolithic S/S samples of BBA/construction aggregates were prepared by adding 5% cement to both types of mixtures, and a normalized diffusion test was carried out to check whether the contaminate load is reduced to levels suitable for use of that material in the construction industry or engineering infrastructures without leading to environmental risks.

## 2. Materials

### 2.1. Biomass Bottom Ashes

In the present work, bottom ashes from three biomass combustion power plants situated in the Andalusia region (in the South of Spain) were studied. Currently, the Renewable Energy Spanish Plan 2011–2020 is being developed, including future goals for the 2020 energy map [18]. Available data indicates that the study area (Andalusia) contains a cultivated area of 8,759,194.93 ha, producing 257.48 MW (Andalusia Energy Agency, 2013). In particular, 1,552,392.92 ha of the total are biomass crops used as fuel by the power plants involved in the present study. Consequently, the potential energy from biomass is 3958 ktep for this region, approximately 40% of which consists of olive trees and products from industries related to the olive agricultural sector (such as olive seed and olive cake). The remaining main biomass crops are pine, holm oak, oak, poplar and eucalyptus (Andalusia Energy Agency, 2013).

All combustion plants belong to Andalusia. To obtain a representative study of the material characteristics, the present work was conducted using 30 samples of BBA obtained from three biomass power plants in the provinces of Málaga, Jaén and Cordoba, and these plants have been named as follows: “Málaga” (BA), “Jaén” (BB) and “Córdoba” (BC). The power capacity of these plants is 33.3 MW, consuming 243,000 tons of biomass annually. All samples were characterized according to the compliance leaching test UNE-EN 12457-3 [19]. The compositions of the mixtures of biomass combusted by the plants included in the present study are listed in Table 1.

Oil cake is the most effective biofuel of all. It is a by-product of olive oil production consisting of seed particles and the fleshy parts of the olive [20]. It contains a large amount of organic substances, of which approximately 4% is oil, which gives oil cake a high potential for energy production [21,22]. This explains why oil cake and olive trees are the biomass components that are burned in higher volumes in all tested plants: approximately 72% of the burning in BA was oil cake, while the BB plant’s combustion was 40% wood biomass (poplar, oak and pine) and BC had a lower amount of oil cake (29%).

After the combustion of the biomass fuel in the high efficiency modern combustion equipment (such as boilers or stoves) of the power plants, mineral constituents of the fuel, in the form of oxides or salts, fall into two components: fly ash, very fine particles that are carried in the flue gases, and bottom ash, larger particles that fall through the grate during combustion [14]. The general characterization of the tested biomass bottom ashes is listed in Table 2 as a general summary of the physical and chemical properties.

After the physical characterization of the BBA samples, it was observed that they were composed of extremely porous particles with rough surface textures. Additionally, the water absorption was measured; for construction materials, it is an important factor to consider because many of the physical parameters of bottom ash are altered in the presence of excess water [23,24]. Water absorption values ranged between 21.75% and 38.74%, notably higher than the absorption levels of aggregates.

### 2.2. Recycled and Natural Aggregates

As has been previously mentioned, the present study provides an environmental assessment of mixtures of BBA/construction aggregates that could be applied as secondary construction materials. For that reason, it is necessary to include the material properties of aggregates that were physically, chemically and environmentally (according to UNE-EN 12457-3 [19]) characterized prior to their environmental characterization. The recycled mixed aggregate (RMA) was manufactured in the treatment plant of Construction and Demolition Wastes (CDW) of the Gecorsa Company, located in Córdoba (Andalusia). The material was composed of 70%–90% concrete and 10%–30% ceramic particles. In this plant, prior to the treatment of the recycled aggregate, the blocks were previously subjected to a cleaning process. Additionally, manual and mechanical selections were performed to separate different compounds from the waste, such as wood, plastic or iron. The production control was performed according to the Standard. The natural aggregate (NA) was extracted from a material accumulation close to road construction work that was chosen due to its high plasticity. Table 3 shows the information concerning the general properties of both aggregates.

From granulometric analysis, it can be stated that the grain size distribution of NA presented a higher fineness than RMA (<0.063 mm). Compared with the BBA, it is observed that the NA and RMA presented higher density values. However, BBA and RMA did not present plasticity, while the natural material NA did present high plasticity values, as indicated in previous paragraphs.

### 2.3. Mixtures of Aggregates and Bottom Ashes

Because the biomass bottom ashes have been demonstrated as unsuitable for use in material construction with bearing capacity, the present research analyzes the behavior of the BBA/construction aggregate mixtures. The dosages of materials in the mixtures come from previous experiments [35] in which it was shown that these dosage percentages are feasible for the use of such mixtures in civil engineering.

The experimental procedure consists of studying the leaching behavior of three BBA samples (representative of each combustion plant) that exhibited a greater release levels of heavy metals. The leaching characterization of the mixtures BBA/NA and BBA/RMA, considered to be granular materials that could potentially be used in civil work, such as roads, was conducted using the percolation test NEN 7343:1994 [36]. According to the previous discussion, the dosage used was 15% BBA and 85% NA or RMA, expressed as the dry weight of the aggregates. 

The last experimental stage of the present research was the solidification/stabilization of both mixtures with 5% cement. These monolithic samples were environmentally characterized according to the Dutch diffusion test EA NEN 7375:2004 [37], and the most significant findings are included in the present work.

**Figure 1:**
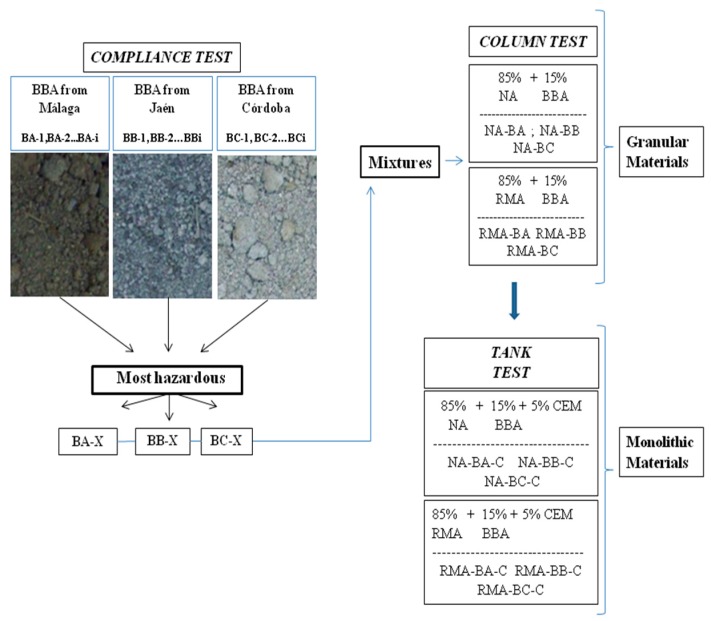
Global materials analyzed in the experimental procedure.

To facilitate understanding of the experimental design developed by the present work, Figure 1 provides a graphical diagram regarding the environmental assessment process performed.

## 3. Experimental Methods

### 3.1. Compliance Test UNE-EN 12457-3:2004 [19]

Compliance testing was conducted to check whether the 30 BBAs satisfy European regulations. To classify those materials according to the EU Landfill Directive, not only heavy metals but also inorganic anions were measured. The UNE-EN 12457-3:2004 [19] procedure consists of a two-step batch leaching test that uses a solution of 175 g of a dry sample of the material, two liquid/solid (L/S) ratios (an L/S of 2 and an L/S of 10) and deionized water as a leaching liquid. This method involves stirring the solution in two steps. In the first step, the solution is shaken for 6 ± 0.5 h with an L/S of 2, and the second step uses the same fraction with stirring of the solution for an additional 18 ± 0.5 h, after adding water to obtain an L/S ratio of 10. In both stages, the samples are left to decant, and the pH, conductivity and temperature are measured. The solution is filtered using a membrane filter (0.45 μm), and a subsample of the leachate was taken for each material for further analysis. 

By this procedure, 30 samples of BBA from the combustion of pine, poplar, oak, eucalyptus, olive and oil cake (composition shown in Table 1) were tested according to the compliance test with the following sample identification code (Table 4).

In addition, the natural aggregate (NA) and the recycled aggregate (RMA) were analyzed by the compliance test, as both materials were mixed with BBA to study how the pollution load of the BBA was affected.

### 3.2. Percolation Test NEN 7343:1994 [36]

The column test described by the standard NEN 7343 is thought to simulate the leaching behavior of a waste material by relating the accumulated released amount of a contaminant, expressed as mg/kg leached, to the liquid/solid ratio. In each column, the leachates were collected at L/S ratios of 0.1, 0.2, 0.5, 1, 2, 5, and 10 L/kg. The translation of the time scale makes it possible to quantify the retention in the matrix, simulating the release progress of a contaminant during the second life cycle of the material [38]. However, the main limitation of this procedure is that laboratory results do not translate directly to field conditions because of factors such as temperature, channeling, the degree and duration of contact with water, ageing effects (carbonation) and others [39,40].

The columns were designed with an inner diameter of 5 cm and a length of 20 cm. The columns were closed with flanges that were sealed. Depending on the material, between 0.5 and 0.7 L was needed to fill the volume of the column. The leachant was deionized water acidified with nitric acid of analytically pure quality to pH = 4 ± 0.1. The pH is not controlled during the test. Therefore, the waste dictates the chemical conditions in the pore-solution. All columns were operated concurrently using multi-channel peristaltic pumps. 

To evaluate the environmental risk of BBAs when they are mixed with other aggregates, nine samples of mixed ashes were tested according to the column test with the following sample identification code (Table 5).

### 3.3. Diffusion Test: NEN 7375:2004 [37]

It is well known that a leaching monolithic under normal conditions of exposure is essentially governed by diffusion [41,42]. Consequently, the most suitable test for laboratory simulation of the material leaching behavior on site is the tank test.

The tank test, as defined by NEN 7375:2004 [37], used concrete cylinders (Ø 10 cm × 12.5 cm) that were cured for 28 days at 20 ± 2 °C and >90% relative humidity (RH), then immersed in a given volume of demineralized water (liquid/solid = 4) and kept in static conditions at a temperature of 20 ± 2 °C.

At the end of each normalized immersion step (6 h and 1, 2.25, 4, 9, 19, 36 and 64 days), the eluate was separated, filtered using a membrane filter (0.45 µm) and stored for further analysis. 

To evaluate the environmental risk of BBAs when they are mixed with other aggregates and cement treated, six samples of mixed-cemented ashes were tested by the diffusion test with the following sample identification code (Table 6).

### 3.4. Chemical Analysis of Eluates

Once each leaching test was completed, filtered and stored leachates corresponding to each step were analyzed by inductively coupled plasma mass spectrometry (ICP-MS) using a Perkin Elmer ELAN DRC-e spectrometer (Perkin Elmer, Waltham, MA, USA). Despite the wide groups of elements measured by the ICP (83 elements), the present study is focused on 12 heavy metals regulated by the Landfill Directive 2003/33 of the European Council regarding the legal criteria and procedures for the acceptance of waste at landfills, the only environmental regulation that is currently accepted by the Spanish Government. Thus, the study includes data concerning the following elements: arsenic (As), lead (Pb), cadmium (Cd), chromium (Cr), copper (Cu), mercury (Hg), nickel (Ni), zinc (Zn), barium (Ba), molybdenum (Mo), selenium (Se) and antimony (Sb).

Test results (mg of leached element per liter of leachate, mg/L) were transformed into accumulated emissions (mg of leached element per kg of aggregate, mg/kg) to compare these values with the limit values established according to the following expression [43]:

(1) *C_x_* (mg *X*/kg aggregate) = (mg *X*/L extracting solution) × (L extracting solution/kg aggregate)

where *C_x_* is the concentration of constituent *X*.

## 4. Results and Discussion

### 4.1. Classification of Materials as a Function of Their Pollutant Potential

Leachate concentrations according to the compliance test are shown in Table 7, Table 8, Table 9 and Table 10. Thirty-two materials (30 BBAs, a natural aggregate (NA) and a recycled aggregate (RMA)) have been classified according to the limit values regulated by the Landfill Directive 2003/33/EC. Green represents inert materials, inert value limits that are exceeded are given in bold and yellow, while non-hazardous limits that are exceeded are underlined and in red.

As and Hg are identified as the most conflictive elements. The concentration of As present was at hazardous levels in 43.3% of the 30 samples, and at non-hazardous and inert levels in the other 56.7%. Hg was detected at hazardous concentration levels in 20% of the samples and at non-hazardous and inert levels in the other 80%.

Other relevant elements are Cr, Ni, Cu, Se and Mo (exceeding the inert limit values in most cases).

Based on the results of the compliance test, due to the high potential contaminants of BBAs, they are unable to be applied in civil engineering as isolated materials. For that reason, the motivation of the present study is to analyze how to reduce the pollutant potential of those products to apply them as secondary materials in construction works.

That is why this study proceeded to analyze mixtures of BBA with other materials to evaluate if reducing the volume of BBA in the sample reduced the contaminant load. 

Regarding the dosage of the mixtures, prior studies were revised. Thus, previous works [35] have proven that mixtures composed of 10%–15% BBA with other aggregates present an appropriate physical and mechanical behavior for use as materials in embankments or road layers [13]. 

To establish the comparison of results between a recycled material and natural aggregate (which should provide better environmental conditions), two groups of samples were prepared in the laboratory: 85% NA with 15% BBA, and 85% RMA with 15% BBA.

Due to the intended application of the materials in civil engineering works (located outdoors and subjected to environmental phenomena), it is necessary that the laboratory study for leaching characterization simulates closely the effect of rain episodes percolating through the granular material and takes place in engineering applications in which this type of material has proven to be suitable and feasible [44,45,46].

**Figure 2:**
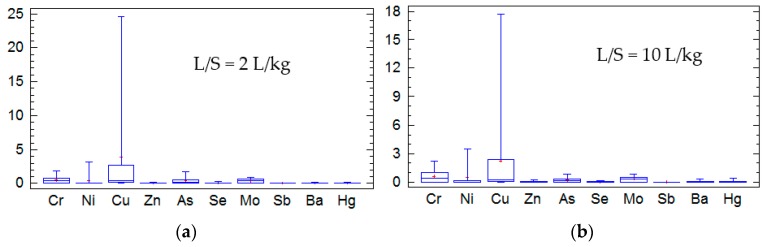
The whisker plot of release (mg/kg) of heavy metals (**a**) L/S = 2 L/kg and (**b**) L/S = 10 L/kg.

Figure 2 shows the results of a statistical analysis performed on data from UNE EN 12457-3 [19] procedure for all of the tested BBA. The analysis is focused on the heavy metals regulated by the UE Landfill Directive. However, Cd and Pb were not included in the analysis because their detected amounts were negligible. The results of the test are shown by means of whisker plots. The first quartile indicates the lowest 25% of the data set, the median separates the lower and upper 50% of the data set, and the lowest 75% represent the fourth quartile. The data from the statistical analysis are summarized in Table 11.

Table 11 shows standard deviations for the most of the studied metals. Only higher standard deviations were observed in the Cu values for L/S = 2 L/kg and L/S = 10 L/kg. 

### 4.2. Data from the Percolation Test

The analysis performed according to the Dutch percolation test, which is itself based on the standard NEN 7343:1994 [36]., was focused on the heavy metals that have been identified as more conflictive according to the obtained results of the prior section: compliance test data (Cr, Ni, Cu, Se, Mo, As and Hg).

In addition, one representative sample was taken from each of the three combustion power plants to perform a representative study of the different BBAs produced in the region of Andalusia. Therefore, from each plant, the BBA with the highest contaminant load according to the data provided in Table 7, Table 8 and Table 9 was chosen in each case: BA-10, BB-3, and BC-7.

**Figure 3:**
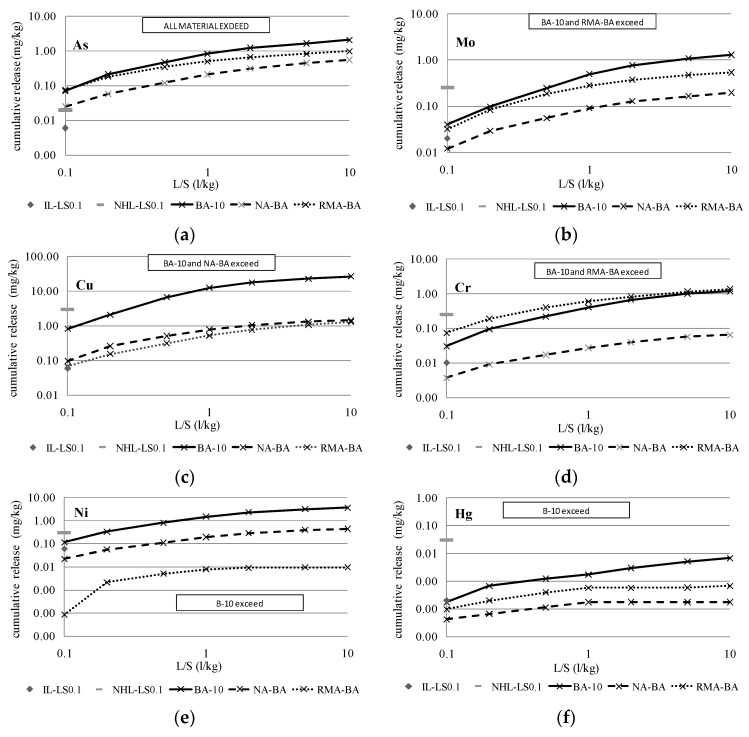
Cumulative release of more conflictive elements for BA-10 and mixtures. IL-LS0.1: Inert limit for column test at L/S = 0.1 L/kg and NHL-LS0.1: Non-hazardous limit for column test at L/S = 0.1 L/kg. (**a**) Cumulative release of As for BA-10 and mixtures; (**b**) Cumulative release of Mo for BA-10 and mixtures; (**c**) Cumulative release of Cu for BA-10 and mixtures; (**d**) Cumulative release of Cr for BA-10 and mixtures; (**e**) Cumulative release of Ni for BA-10 and mixtures; (**f**) Cumulative release of Hg for BA-10 and mixtures

**Figure 4:**
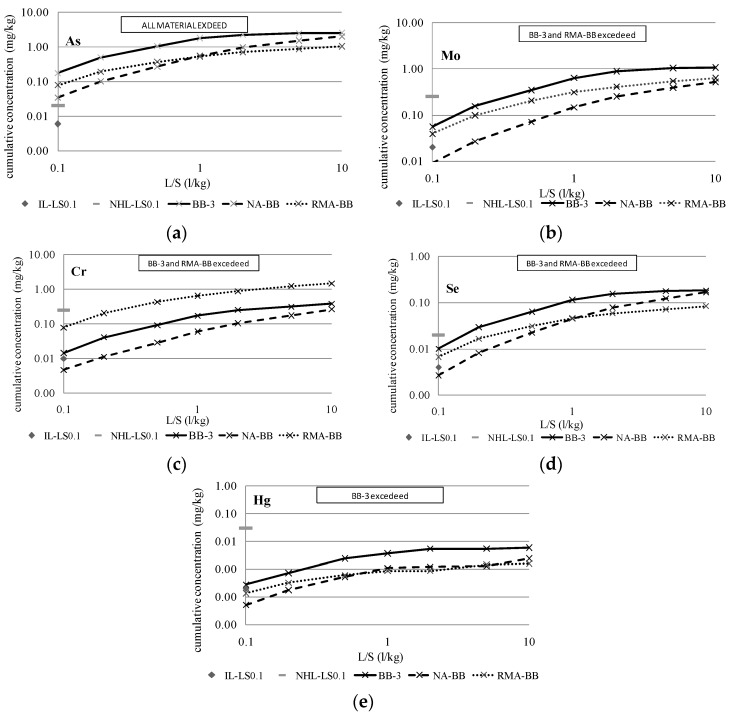
Cumulative release of more conflictive elements for BB-3 and mixtures. IL-LS0.1: Inert Limit for column test at L/S = 0.1 L/kg and NHL-LS0.1: Non-hazardous limit for column test at L/S = 0.1 L/kg. . (**a**) Cumulative release of As for BB-3 and mixtures; (**b**) Cumulative release of Mo for BB-3 and mixtures; (**c**) Cumulative release of Cr for BB-3 and mixtures; (**d**) Cumulative release of Se for BB-3 and mixtures; (**e**) Cumulative release of Hg for BB-3 and mixtures.

**Figure 5:**
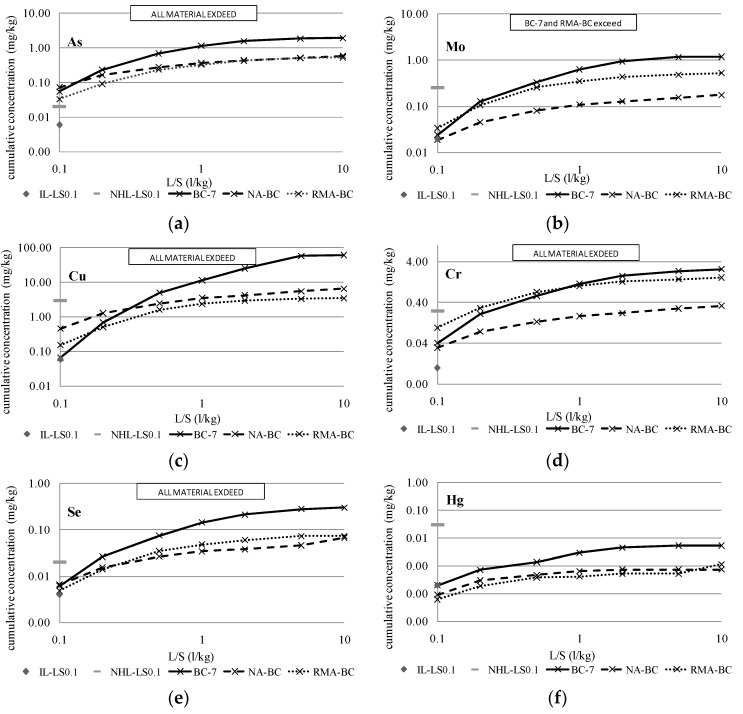
Cumulative release of more conflictive elements for BC-7 and mixtures. IL-LS0.1: Inert limit for column test at L/S = 0.1 L/kg and NHL-LS0.1: Non-hazardous limit for column test at L/S = 0.1 L/kg. (**a**) Cumulative release of As for BC-7 and mixtures; (**b**) Cumulative release of Mo for BC-7 and mixtures; (**c**) Cumulative release of Cu for BC-7 and mixtures; (**d**) Cumulative release of Cr for BC-7 and mixtures; (**e**) Cumulative release of Se for BC-7 and mixtures; (**f**) Cumulative release of Hg for BC-7 and mixtures.

To compare the reduction of the contaminant load between the samples composed of 100% BBA (BA-10, BB-3 and BC-7) and the mixtures with 15% BBA, Figure 3, Figure 4 and Figure 5 show the cumulative release curves obtained by the percolation test, including the curves of the mixtures with aggregates (NA-BA, RMA-BA, NA-BB, RMA-BB, NA-BC and RMA-BC).

All graphics include the inert legal limit value for the percolation leaching test data imposed by the Landfill Directive at the L/S of 0.1 L/kg (IL-LS0.1, grey rhombus) and the non-hazardous limit (NHL-LS0.1, grey short line). Thus, the samples that have exceeded this limit are marked, being the material (BBA or mixtures) classified as non-hazardous. According to the results, the release of As has exceeded the inert and non-hazardous limits in all samples, with the BBA and mixtures classified as non-hazardous materials according to the percolation data.

Regarding the elements Cr, Ni, Cu, Se and Mo, which were detected as more contaminant-filled by the compliance test in the BBA samples based on the column tests performed for the mixtures of BBA with aggregates, their pollutant potential is even higher and, in most of cases, the inert limit is obviously exceeded; the highest release was obtained for the three BBAs, BA-10, BB-3 and BC-7. The patterns described during the percolation test and, as a consequence, the cumulative percolation curves, were quite similar in both BBA and the mixtures. However, as expected, the cumulative releases of BA-10, BB-3 and BC-7 were the highest of the total data represented. Obviously, and according to the results illustrated in Table 10, the mixtures of BBA with NA presented the lowest release levels, while the RMA mixtures showed higher pollutant levels.

The most noteworthy difference between both types of mixtures is observed in the release pattern of Cr: in all cases, the percolation curves of Cr in RMA mixtures were markedly higher than NA mixtures and BBA curves. That difference is caused by the high content of Cr in the ceramic particles present in recycled aggregates from construction and demolition waste (CDW) [47].

After the discussion of the percolation data, the reduction of the pollution load of hazardous BBAs (BA-10, BB-3 and BC-7) following their combination with aggregates can be confirmed. This reduction implies the possibility of reuse as secondary materials in construction works rather than discarding them and depositing them in landfills (which favors a negative environmental impact and does not contribute to increasing the value of industrial by-products).

Accordingly, the environmental assessment has confirmed the feasibility of the combination of both materials for its use in civil works.

To expand the scope of the study within the framework of civil engineering, the following section analyses the pollution potential of BBA mixtures treated with cement. Previous studies have proven their viability [44]. For that, the Dutch diffusion test was performed according to the standard EA NEN-7375:2004 [37] for the monolithic samples prepared in the laboratory (six BBA mixtures of RMA and NA with 5% cement).

In order to evaluate the immobilization degree reached with the experimental procedure developed, Table 12 shows the percentage of reduction of the release levels measured in the BBA samples (BA10, BB3 and BC7, representative of all the studied combustion plants) compared with the release levels obtained for the mixtures with the natural material NA and the recycled aggregate RMA. For establishing the comparison and calculation of the ratio, the last data (cumulative release at L/S of 10 L/kg) of the column test was considered.

The immobilization ratio is shown in Table 12, calculated according to Equation (2):

(2) $$\%\text{reduction}=\;\left(\frac{\text{Bi}-\text{AGi}}{\text{AGi}}\right)\times 100$$

where Bi: Release level of each element for biomass bottom ashes: BA10 for first plant; BB3 for second plant or BC7 for third plant; AGi: Release level of each element for mixtures of 85% of natural aggregate or recycled mixed aggregates + 15% of BBA: NA-BA; RMA-BA; NA-BB; RMA-BB; NA-BC; RMA-BC.

According to data, in general a reduction of the pollutant levels is observed for all mixtures and all elements. The highest immobilization is observed in the mixture of BA10 with the natural aggregate NA (BA10 & NA-BA). The lowest immobilization ratio was observed for the mixtures of NA and RMA with the BB3 (BB3 & NA-BB and BB3 & RMA-BB).

### 4.3. Data from the Diffusion Test

After verifying the reduction of the pollution load of the BBA after being mixed with RMA and NA, this investigation advanced a step further by analyzing the behavior of the stabilized/solidified mixtures. The tank test or diffusion process through the Dutch EA NEN 7375:2004 [37] was performed for the monolithic cylindrical samples (Ø 10 cm × 12.5 cm) prepared in the laboratory with 5% cement.

This percentage was chosen because it is the most common for civil engineering materials used as road base and sub-base dosage [48,49]. The objective is to prove the reduction of the pollution load of granular materials after their treatment with cement. Thus, the environmental benefit will be shown in addition to checking that these materials can be applied as construction materials (which was previously demonstrated by other researchers).

**Figure 6:**
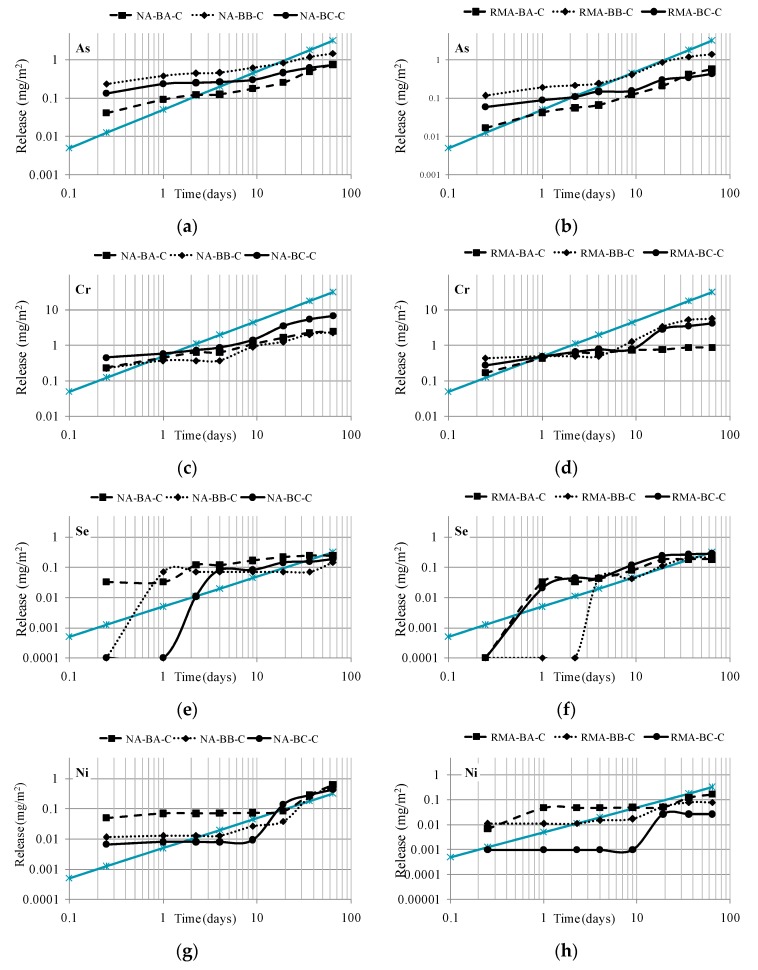
Diffusion release curves of more conflictive elements for monolithic mixtures. (**a**) Diffusion release curves of As for monolithic mixtures manufactured with NA; (**b**) Diffusion release curves of As for monolithic mixtures manufactured with RMA; (**c**) Diffusion release curves of Cr for monolithic mixtures manufactured with NA; (**d**) Diffusion release curves of Cr for monolithic mixtures manufactured with RMA; (**e**) Diffusion release curves of Se for monolithic mixtures manufactured with NA; (**f**) Diffusion release curves of Se for monolithic mixtures manufactured with RMA; (**g**) Diffusion release curves of Ni for monolithic mixtures manufactured with NA; (**h**) Diffusion release curves of Hg for monolithic mixtures manufactured with RMA.

Figure 6 shows the diffusion curves of the most conflictive elements detected in 6 monolithic samples cemented with CEM II/BL 32.5.

Figure 6 illustrates the diffusion release curves of the elements As, Cr, Se and Ni. Additionally, a slope of 1:0.5 (the grey line) is represented graphically to facilitate the identification of the mechanisms that govern the release. Previous researchers have proven that pure diffusion-controlled release implies a 1:0.5 slope [50]. Additionally, other patterns can be observed in the graphic, such as the depletion of elements that describe a flat line during different periods of time.

The patterns of diffusion have been described according to Figure 6. The element present at the highest levels in the tested BBA was As. It presented mixed behavior: in the first stages, until the fourth day, the release was very stable and there were hardly any significant differences with time. However, from the fourth day on, the diffusion curve of As was parallel to the slope 1:0.5 and, therefore, the governing mechanism was the diffusion of the element.

Cr presents a similar pattern to that of As, but Cr showed depletion in the last days of the test. It must be noted that for the monolithic samples (after the treatment with cement of the RMA and NA mixtures), no differences were observed between the release diffusion curves of both mixtures. This could imply/prove that the treatment with cement is causing the isolation of the material matrix, and that the expected higher Cr release in the mixture due to the presence of ceramic particles in RMA is not produced in the monolithic samples. This phenomenon occurred in the granular samples RMA-BA, RMA-BB, RMA-BC due to the absence of isolation of their internal matrix.

From the fourth day on, Se exhibited a curve parallel to the 1:0.5 slope; this behavior was more evident in the RMA mixtures. Finally, Ni presented a flat phase for most of the test, which demonstrated its low solubility. Only from day 19 was its release curve parallel to the diffusion slope.

### 4.4. Comparison between the Percolation and Diffusion Tests

The present section is focused on evaluating the reduction of the contaminant load of the more contaminated BBAs: BA-10, BB-3 and BC-7.

The data treatment was performed for Section 4.3 and Section 4.4 as follows. The standard EA NEN 7375:2004 [37] contains, in Section “8. Calculation”, the formula for the calculation of the measured leaching per fraction:

(3) $$E_{i}^{*}=\;\frac{C_{i\;}\times \;V}{f\;\times \;A}$$

where Ei* is the measured leaching of a component in fraction *i* in mg/m^2^. *C_i_* is the concentration of the component in fraction *i* in µg/L. *V* is the volume of the eluate in L. *A* is the surface area of the test piece in m^2^. *f* is a conversion factor: 1000 µg/mg.

The measured cumulative leach $\varepsilon_{n\;}^{*}$ in each period (*n* = 1 to *N*) was calculated by:

(4) $$\varepsilon_{n\;}^{*}=\;\sum_{i=1}^{n}E_{i}^{*}\;\;\text{for}\;\;n=1\;\text{to}\;N$$

where $\varepsilon_{n\;}^{*}$ is the measured cumulative leaching of a component for period *n* consisting of fractions *i* = 1 to *n* in mg/m^2^. E_i_* is the measured leaching of a component in fraction *i* in mg/m^2^. *N* is the number of periods equal to the amount of specified replenishment time (*N* = 8).

According to Equation (3), the cumulative curves were obtained and represented in Figure 5. To compare the data of the percolation test with the diffusion curves, Equation (4) was applied. Thus, concentrations expressed in µg/L were transformed into mg/m^2^, and the data are presented in Figure 6. 

**Figure 7:**
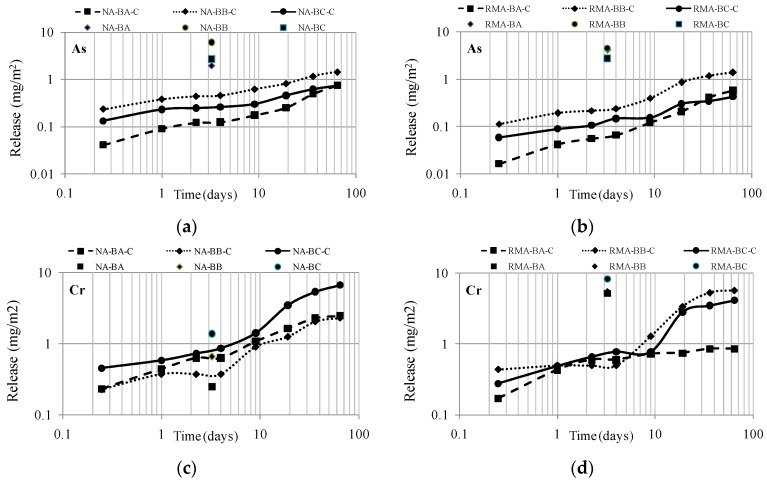
Superimposition of diffusion release curves and percolation data for As and Cr. (**a**) Superimposition of diffusion release curves and percolation data for As (mixtures with NA); (**b**) Superimposition of diffusion release curves and percolation data for As (mixtures with RMA); (**c**) Superimposition of diffusion release curves and percolation data for Cr (mixtures with NA); (**d**) Superimposition of diffusion release curves and percolation data for Cr (mixtures with RMA).

To compare the results of both tests, the leachate value of the percolation test at an L/S of 2 L/kg is assumed. This value was transformed into the unit time to superimpose the data on the diffusion curves (Figure 7).

The L/S ratio depends directly on the volume (expressed in cm^3^) and the dry mass (d.m.) of the sample (kg d.m.) because the process described by the standard EA NEN 7375:2004 [37] requires a constant water flow of 0.3 mL/min. Starting from these variables, it was decided to adopt the data at L/S = 2 L/kg (being the commonly used test leaching compliance) to superimpose the diffusion and percolation data. According to the percolation test procedure, this L/S ratio is reached after 77.78 h, which is 3.25 days, as observed in the x-axis of the graphs of Figure 7.

As a function of the results obtained, only the two more conflicting elements, from the environmental point of view, As and Cr, have been chosen to be studied in depth.

Regarding the release of As in leachates, identical diffusion curves are obtained in both mixtures. Thus, no differences are observed between the mixtures with the natural aggregate, NA, and the recycled one, RMA. Comparison highlights the significant decrease in As released by the monoliths (NA-BA-C, NA-BB-C and NA-BC-C) compared to the high value of metal released from the samples in their granular form (NA-BA, NA-BB and NA-BC). The data are consistent with previous research studies [51].

Regarding leaching behavior of Cr, a different response was observed depending on the type of aggregate used for the mixture elaboration. The fundamental difference lies in the composition of the leachate of NA and RMA (see Table 10). While the natural aggregate released only 0.001 mg/kg and 0.012 mg/kg (at an L/S of 2 and 10, respectively), the recycled construction aggregate released 0.265 mg/kg and 0.374 mg/kg, respectively.

Therefore, when preparing mixtures to be tested by the percolation leaching method, the contaminant load is conditioned by the aggregate used. Thus, when the data of the tank and column are overlapped in Figure 7, it is observed that, in mixtures of BBA and RMA, the reduction of pollution potential after the S/S treatment with cement was remarkable compared to the poor reduction observed in the mixture of BBA and NA after the S/S treatment with cement. The results are logical and coherent with expectations, as a comparison confirms that no contamination reduction occurs in materials that presented low Cr levels in the source material (NA, see Table 10).

The effectiveness of the S/S treatment with cement for materials with high pollutant loads (BBA samples BA-10, BB-3 and BC-7, classified as hazardous materials) can be based on two observations: (1) The release of mixtures of the granular materials RMA-BA, RMA-BB and RMA-BC were much higher than those obtained by the monolithic RMA-BA-C, RMA-BB-C and RMA-BC-C; (2) The diffusion curves of the mixtures of BBA manufactured with aggregates with different pollutant loads presented similar diffusion curves and similar release levels after the S/S treatment.

## 5. Conclusions

The conclusions of the present work can be summarized in three bullet points, corresponding to the three experimental stages developed:

- The compliance test data revealed a contaminant potential in some of the samples analyzed. Thus, 37% of tested BBAs were classified as inert, 13% as non-hazardous, and 50% as hazardous, which confirms that they are a material unsuitable for application as an isolated aggregate in any application of civil engineering. The heavy metals released in higher levels were, in order of relevance, As, Hg, Cr, Ni, Cu, Se and Mo. According to that, the second stage consisted of analyzing the mixtures of the most contaminated BBAs (BA, BB and BC) with other construction aggregates, to evaluate whether the volume reduction of BBA in samples implies a reduction of the contaminant load.
- The percolation test provided the cumulative curves of all elements for all mixtures. According to the registered data and again applying the legal limit values imposed by the Landfill Directive, it was observed that after mixture, even for the most hazardous BBA with the NA and the RMA, the contaminant loads of the aggregates were reduced. The third stage of the investigation was the evaluation of the leaching behavior of the S/S mixtures with the objective of proving the pollution load reduction of aggregates and BBA after their treatment with cement.
- Data from the tank test performed on monolithic samples indicated that no differences were observed between the release diffusion curves of both mixtures types BBA/NA and BBA/RMA, despite their different chemical compositions. This demonstrates the effectiveness of the S/S treatment for materials that present a high pollution potential (as occurred with the BBAs in the study, as they were classified as hazardous wastes). According to the findings, secondary materials such as biomass bottom ash can be reused from an environmental point of view, as long as adequate management of these materials is performed by engineers, constructors or plant managers. Thus, the present work proposes a solution that implies an environmental benefit to those agents, in addition to checking the samples (as unbound aggregates and as S/S mixtures), with the potential for being applied as construction materials during their second life cycle.

## Figures and Tables

**Table 1:** Average values composition of biomass combusted by the studied plants.

Composition of Biomass (%)			
Plants	Wood Waste (Pine, Poplar, Oak, Eucalyptus)	Olive Tree	Oil Cake
Málaga (BA)	22	6	72
Jaén (BB)	40.5	17	42.5
Córdoba (BC)	27	44	29

**Table 2:** Mean values of different physical and chemical parameters of tested biomass bottom ash (BBA).

Properties		BA	BB	BC	Test Method
Density-SSD (kg/m^3^)	Size 0–4 mm	1.70	2	1.72	UNE-EN 1097-01 [25]
Water absorption (%)	Size 0–4 mm	29.40	21.75	38.74	UNE-EN 1097-01 [25]
Friability ratio (%)		28	30	33	UNE 83-115 [26]
Plasticity		Non plastic	Non plastic	Non plastic	UNE 103103 & UNE 103104 [27,28]
Chlorides		-	-	0.15	UNE-EN 1774-1 [29]
Elemental content (%)	Si	24.68	25.06	24.23	UNE 80-215 [30]
	Ca	11.03	15.52	13.85	
	K	11.11	15.22	11.55	
	Mg	2.10	3.16	2.22	
	Fe	1.30	1.86	1.39	
	Al	0.53	0.59	0.51	
	Na	0.43	0.42	0.46	
	Ti	0.16	0.20	0.17	
Organic matter (%)		12.3	4.05	3.67	UNE 103204 [31]
Water-soluble sulfate (%SO_3_)		0.27	0.36	0.30	UNE-EN 1744-1 [32]
Acid-soluble sulfate (%SO_3_)		0.32	0.38	0.35	UNE-EN 1744-1 [33]

**Table 3:** Properties of the recycled and natural aggregates.

Properties		Test Method	Recycled Mixed Aggregates (RMA)	Natural Aggregate (NA)
Density-SSD (g/cm^3^)	Size 0–4 mm	UNE-EN 1097-01 [25]	2.04	2.56
	Size 4–31.5 mm		2.11	2.6
Water absorption (%)	Size 0–4 mm	UNE-EN 1097-01 [25]	9.42	2.52
	Size 4–31.5 mm		10.98	1.9
Plasticity		UNE 103103 & UNE 103104 [27,28]	6.49	Non plastic
Water-soluble sulfate content		UNE-EN 1744-1 [32]	0.5	<0.01
Acid-soluble sulfate content		UNE-EN 1744-1 [33]	0.7	<0.01
Particle size distribution (mm)	31.5	UNE-EN 933-2 [34]	100	100
	20		94	90
	8		54	66
	4		38	50
	2		29	42
	0.5		17	32
	0.063		4	20

**Table 4:** Sample ID code for BBA samples analyzed by the compliance test UNE-EN 12457-3:2004.

ID Code for BBA Tested Samples	Biomass Power Plant of Origin
BAi (BA-1, BA-2, BA-3, …, BA-10)	Málaga (BA)
BBi (BB-1, BB-2, BB-3, …, BB-10)	Jaen (BB)
BCi (BC-1, BC-2, BC-3, …, BC-10)	Córdoba (BC)

**Table 5:** Sample ID code for mixed BBA analyzed by the column test NEN 7343:1994.

ID Code for BBA Tested Samples	Description of the Granular Materials
BA-10, BB-7, BC-3	100% of BBA from Málaga, Jaén and Córdoba combustion plants
NA-BA, NA-BB, NA-BC	Mixtures of 85% of natural aggregate + 15% of BBA
RMA-BA, RMA-BB, RMA-BC	Mixtures of 85% of recycled aggregate + 15% of BBA

**Table 6:** Sample ID code for mixed BBA analyzed by the diffusion test EA NEN 7375:2004 [37].

ID Code for BBA Tested Samples	Description of Monolithic Samples
NA-BA-C, NA-BB-C, NA-BC-C	Mixtures of 85% of natural aggregate + 15% of BBA cemented with 5% cement CEM II
RMA-BA-C, RMA-BB-C, RMA-BC-C	Mixtures of 85% of recycled aggregate + 15% of BBA cemented with 5% cement CEM II

**Table 7:** Leachate concentrations (mg/kg) for Malaga BBA by UNE EN 12457-3 [19].

Element	BA-1		BA-2		BA-3		BA-4		BA-5		BA-6		BA-7		BA-8		BA-9		BA-10	
	L/S 2	L/S 10	L/S 2	L/S 10	L/S 2	L/S 10	L/S 2	L/S 10	L/S 2	L/S 10	L/S 2	L/S 10	L/S 2	L/S 10	L/S 2	L/S 10	L/S 2	L/S 10	L/S 2	L/S 10
Cr	0.01	0.00	0.01	0.00	0.01	0.00	0.56	0.71	0.01	0.00	0.01	0.00	0.10	0.15	1.57	1.48	0.42	0.48	0.68	0.73
Ni	0.10	0.03	0.05	0.01	0.08	0.02	2.84	3.49	0.05	0.01	0.04	0.01	0.27	0.26	3.20	3.27	2.30	2.51	2.72	2.99
Cu	0.45	0.17	0.25	0.05	0.78	0.19	18.33	17.69	0.61	0.14	0.39	0.09	3.18	3.52	22.72	14.94	19.68	1.99	24.59	5.05
Zn	0.00	0.00	0.00	0.00	0.00	0.00	0.03	0.23	0.00	0.00	0.00	0.00	0.01	0.03	0.03	0.06	0.01	0.10	0.03	0.09
As	0.01	0.00	0.00	0.00	0.01	0.00	0.52	0.25	0.01	0.00	0.01	0.00	0.01	0.00	*0.98*	0.40	*0.70*	0.38	*1.27*	0.53
Se	0.00	0.00	0.00	0.00	0.00	0.00	0.13	0.08	0.00	0.00	0.00	0.00	0.01	0.00	0.10	0.00	0.13	0.02	0.14	0.02
Mo	0.00	0.00	0.01	0.00	0.00	0.00	0.35	0.32	0.00	0.00	0.01	0.00	0.25	0.24	0.65	0.50	0.76	0.36	0.88	0.57
Sb	0.00	0.00	0.00	0.00	0.00	0.00	0.02	0.03	0.00	0.00	0.00	0.00	0.00	0.00	0.00	0.01	0.01	0.02	0.01	0.01
Ba	0.00	0.00	0.00	0.00	0.00	0.00	0.02	0.05	0.00	0.00	0.00	0.00	0.03	0.20	0.03	0.04	0.02	0.05	0.05	0.09
Hg	0.00	0.00	0.00	0.00	0.00	0.00	0.00	0.10	0.00	0.00	0.00	0.00	0.00	0.06	0.00	0.01	*0.12*	0.07	*0.16*	0.10
classification	IL	IL	IL	IL	IL	IL	HL	NHL	IL	IL	IL	IL	NHL	NHL	HL	NHL	HL	NHL	HL	NHL

Notes: IL: inert limit; HL: hazardous limit; NHL: non-hazardous limit.

**Table 8:** Leachate concentrations (mg/kg) for Jaén BBA by UNE EN 12457-3 [19].

Element	BB-1		BB-2		BB-3		BB-4		BB-5		BB-6		BB-7		BB-8		BB-9		BB-10	
	L/S 2	L/S 10	L/S 2	L/S 10	L/S 2	L/S 10	L/S 2	L/S 10	L/S 2	L/S 10	L/S 2	L/S 10	L/S 2	L/S 10	L/S 2	L/S 10	L/S 2	L/S 10	L/S 2	L/S 10
Cr	0.02	0.01	0.30	0.36	0.36	0.46	0.01	0.00	0.78	1.02	0.03	0.01	0.75	0.86	1.31	1.50	0.34	0.39	0.52	0.59
Ni	0.00	0.00	0.03	0.03	0.03	0.02	0.01	0.00	0.02	0.02	0.00	0.00	0.03	0.05	0.01	0.01	0.02	0.03	0.10	0.09
Cu	0.02	0.00	0.06	0.18	0.14	0.13	0.22	0.03	0.37	0.28	0.07	0.01	0.10	0.55	0.17	0.13	0.05	0.44	2.69	2.92
Zn	0.00	0.00	0.02	0.03	0.03	0.03	0.00	0.00	0.06	0.10	0.00	0.00	0.06	0.06	0.02	0.08	0.06	0.13	0.02	0.05
As	0.01	0.00	0.19	0.18	0.51	0.31	0.08	0.01	1.74	0.83	0.02	0.00	0.28	0.18	0.57	0.36	1.59	0.72	0.45	0.28
Se	0.00	0.00	0.06	0.05	0.07	0.00	0.01	0.00	0.09	0.05	0.01	0.00	0.09	0.02	0.15	0.06	0.12	0.02	0.08	0.00
Mo	0.02	0.00	0.26	0.28	0.40	0.41	0.03	0.01	0.71	0.68	0.02	0.00	0.32	0.29	0.45	0.39	0.72	0.65	0.57	0.53
Sb	0.00	0.00	0.01	0.03	0.02	0.04	0.00	0.00	0.03	0.04	0.00	0.00	0.01	0.01	0.01	0.02	0.01	0.01	0.01	0.03
Ba	0.00	0.00	0.01	0.02	0.01	0.02	0.00	0.00	0.03	0.05	0.00	0.00	0.01	0.03	0.01	0.03	0.03	0.04	0.01	0.04
Hg	0.00	0.00	0.00	0.05	0.06	0.05	0.00	0.00	0.01	0.01	0.00	0.00	0.00	0.00	0.00	0.13	0.00	0.00	0.00	0.04
classification	IL	IL	NHL	NHL	HL	NHL	IL	IL	HL	NHL	IL	IL	NHL	NHL	HL	NHL	HL	NHL	HL	HL

**Table 9:** Leachate concentrations (mg/kg) for Córdoba BBA by UNE EN 12457-3 [19].

Element	BC-1		BC-2		BC-3		BC-4		BC-5		BC-6		BC-7		BC-8		BC-9		BC-10	
	L/S 2	L/S 10	L/S 2	L/S 10	L/S 2	L/S 10	L/S 2	L/S 10	L/S 2	L/S 10	L/S 2	L/S 10	L/S 2	L/S 10	L/S 2	L/S 10	L/S 2	L/S 10	L/S 2	L/S 10
Cr	0.05	0.01	0.72	0.89	0.03	0.01	0.96	1.19	1.09	1.17	0.06	0.01	1.92	2.19	1.64	1.92	0.24	0.23	1.31	1.45
Ni	0.00	0.00	0.11	0.12	0.01	0.00	0.12	0.14	0.62	0.73	0.00	0.00	0.03	0.03	0.01	0.01	0.01	0.01	0.05	0.05
Cu	0.15	0.02	2.51	2.37	0.18	0.03	3.22	2.82	9.76	9.52	0.16	0.03	1.24	1.04	0.29	0.16	0.33	0.28	2.22	1.88
Zn	0.00	0.00	0.04	0.04	0.00	0.00	0.05	0.05	0.04	0.09	0.00	0.00	0.15	0.09	0.10	0.08	0.01	0.04	0.03	0.07
As	0.01	0.00	0.82	0.41	0.04	0.01	1.34	0.62	0.53	0.28	0.01	0.00	0.49	0.25	0.07	0.02	0.03	0.03	0.43	0.25
Se	0.00	0.00	0.22	0.14	0.01	0.00	0.28	0.14	0.16	0.08	0.01	0.00	0.13	0.05	0.09	0.03	0.00	0.00	0.07	0.00
Mo	0.01	0.00	0.85	0.83	0.02	0.01	0.86	0.81	0.44	0.43	0.03	0.01	0.68	0.59	0.35	0.34	0.05	0.05	0.46	0.45
Sb	0.00	0.00	0.02	0.03	0.00	0.00	0.01	0.02	0.01	0.01	0.00	0.00	0.01	0.01	0.00	0.00	0.00	0.01	0.01	0.01
Ba	0.00	0.00	0.02	0.02	0.00	0.00	0.02	0.02	0.07	0.18	0.00	0.00	0.18	0.35	0.06	0.19	0.00	0.01	0.01	0.03
Hg	0.00	0.00	0.20	0.17	0.00	0.00	0.01	0.18	0.00	0.00	0.00	0.00	0.01	0.35	0.01	0.43	0.01	0.00	0.04	0.00
classification	IL	IL	HL	NHL	IL	IL	HL	NHL	HL	HL	IL	IL	HL	HL	NHL	HL	NHL	IL	HL	NHL

**Table 10:** Leachate concentrations (mg/kg) for aggregates by UNE EN 12457-3 [19].

Aggregate	Ratio	Cr	Ni	Cu	Zn	As	Se	Mo	Sb	Ba	Hg
NA	L/S 2	0.00	0.00	0.01	0.01	0.00	0.00	0.02	0.00	0.04	0.00
	L/S 10	0.01	0.00	0.02	0.05	0.02	0.00	0.04	0.00	0.13	0.00
RMA	L/S 2	0.27	0.01	0.01	0.02	0.00	0.01	0.15	0.01	0.10	0.00
	L/S 10	0.37	0.01	0.02	0.24	0.01	0.00	0.20	0.02	0.32	0.00

Note: Cd and Pb were negligible and inferior to the detection limit.

**Table 11:** Summary of statistical data for leaching data according to UNE EN 12457-3 [19].

Data for L/S Ratio of 2 L/kg					
Element	Minimum	Maximum	Average	Variance	Typical Deviation
Cr	0.010	1.920	0.527	0.323	0.568
Ni	0.000	3.200	0.429	0.897	0.947
Cu	0.020	24.590	3.831	53.156	7.291
Zn	0.000	0.150	0.027	0.001	0.034
AS	0.000	1.740	0.424	0.261	0.511
Se	0.000	0.280	0.072	0.005	0.074
Mo	0.000	0.880	0.339	0.099	0.314
Sb	0.000	0.030	0.007	0.000	0.008
Ba	0.000	0.180	0.021	0.001	0.035
Hg	0.000	0.200	0.021	0.002	0.050
**Data for L/S Ratio of 10 L/kg**					
**Element**	**Minimum**	**Maximum**	**Average**	**Variance**	**Typical Deviation**
Cr	0.000	2.190	0.594	0.413	0.643
Ni	0.000	3.490	0.465	1.113	1.055
Cu	0.000	17.690	2.222	18.900	4.347
Zn	0.000	0.230	0.048	0.003	0.052
As	0.000	0.830	0.210	0.057	0.239
Se	0.000	0.140	0.025	0.002	0.040
Mo	0.000	0.830	0.292	0.074	0.273
Sb	0.000	0.040	0.011	0.000	0.013
Ba	0.000	0.350	0.049	0.006	0.079
Hg	0.000	0.430	0.058	0.011	0.105

**Table 12:** Reduction percentage of release levels calculated according to the column test data.

Element	% Reduction BA10 & NA-BA	% Reduction BA10 & RMA-BA	% Reduction BB3 & NA-BB	% Reduction BB3 & RMA-BB	% Reduction BC7 & NA-BC	% Reduction BC7 & RMA-BC
As	73.5	53	21.1	58.9	69.5	72
Ba	25.7	63.3	2.6	2.8	67	74.9
Cd	73.4	70.8	24.8	56.2	67.6	75.4
Cr	94.4	4.4	30.5	4.1	87.4	37.9
Cu	94.6	95.1	72.1	95.2	89.4	94.3
Hg	97.4	90	59	73	86.1	78.5
Mo	84.9	58.5	51.4	41.4	85.1	55.8
Ni	87.9	99.7	2.03	64.3	86.8	95.7
Pb	94.4	94.6	92.4	97.9	97.8	95.7
Sb	66.6	44.8	37.7	5.3	38	2.7
Se	80.3	78.6	8.1	54.1	77.7	75.7
Zn	87.2	82.1	55.4	93.8	94.1	98.6
